# Supplementary material for: A Novel m7G-Related Genes-Based Signature with Prognostic Value and Predictive Ability to Select Patients Responsive to Personalized Treatment Strategies in Bladder Cancer
Source: Cancers (Basel). 2022 Oct 29;14(21):5346. doi: 10.3390/cancers14215346 (PMC9656096; doi:10.3390/cancers14215346)
Supplement: Supplementary file 1 [file cancers-14-05346-s001.zip › Supplementary Figures.pdf]

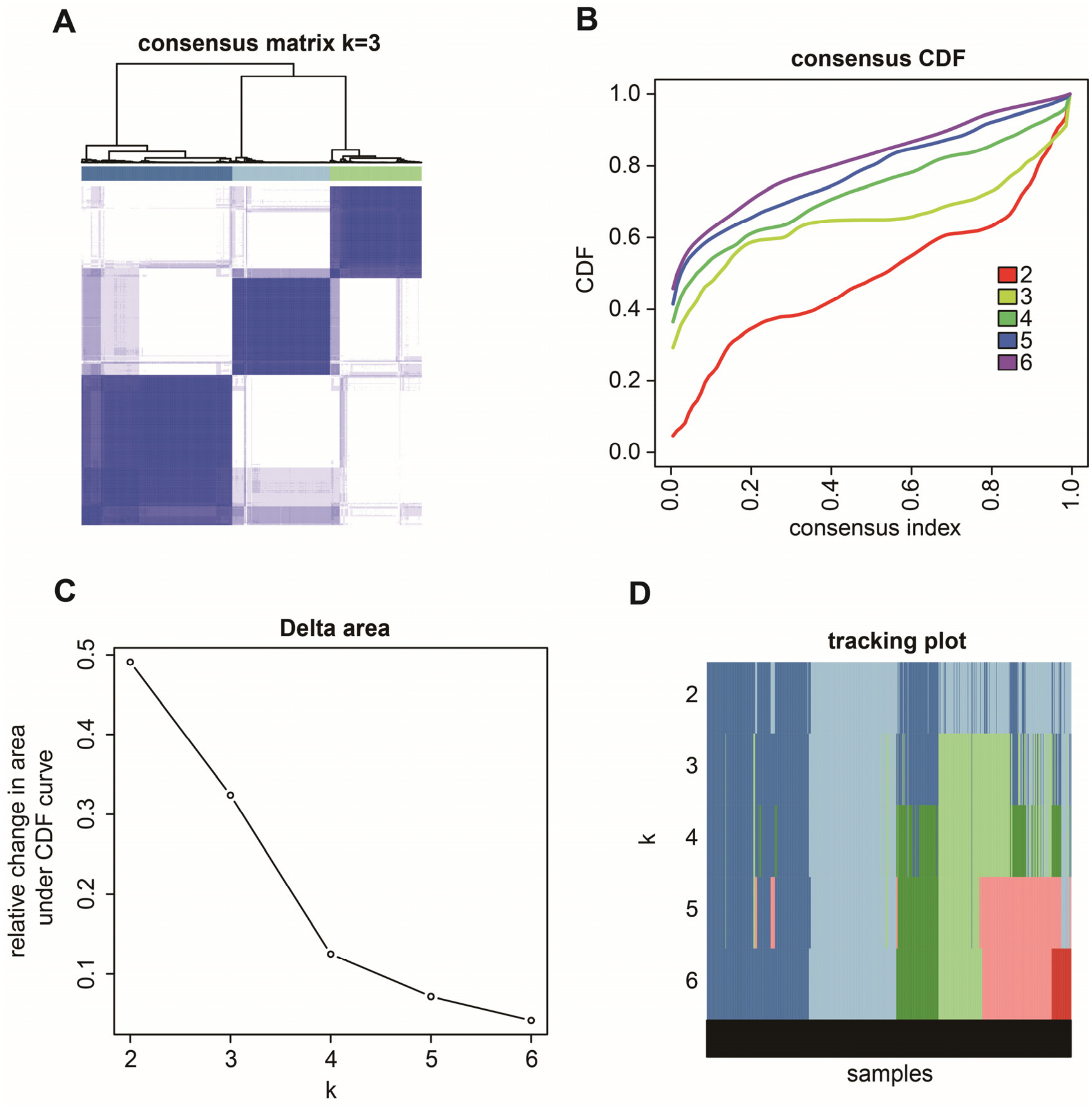

Figure S1. Consensus unsupervised clustering of 760 BLCA patients based on 18 m7G-related genes. **A** Consensus matrixes of 760 BLCA patients when k=3. **B** The correlation between consensus index and CDF when k = 2-6. **C** The relative change under the CDF curve when k = 2-6. **D** The tracking plot of 760 patients when k=2-6.

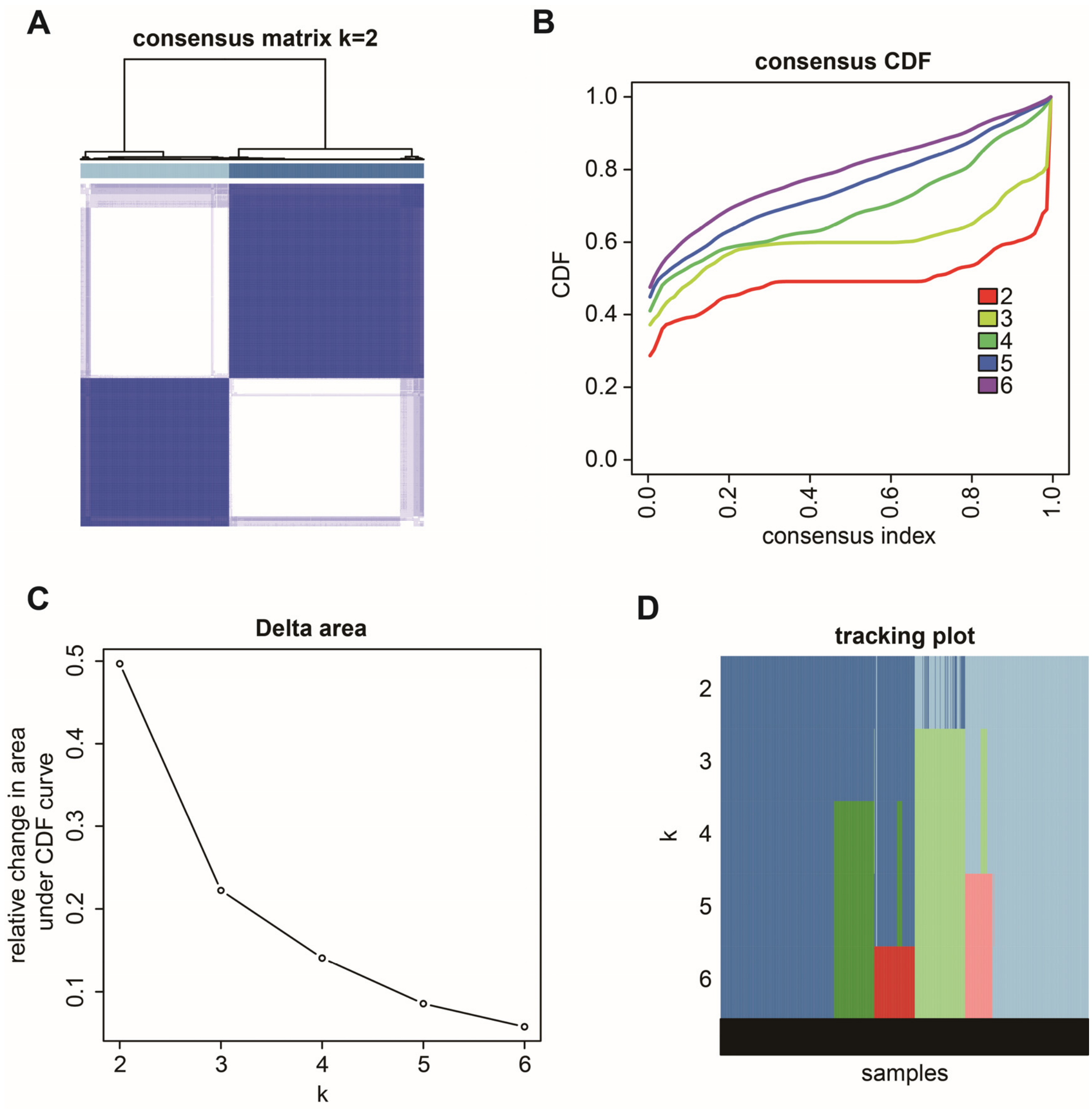

Figure S2. Consensus unsupervised clustering of 760 BLCA patients based on 1108 differentially expressed genes relevant to three m7G-related clusters. **A** Consensus matrixes of 760 BLCA patients when k=2. **B** The correlation between consensus index and CDF when k = 2-6. **C** The relative change under the CDF curve when k = 2-6. **D** The tracking plot of 760 patients when k=2-6.

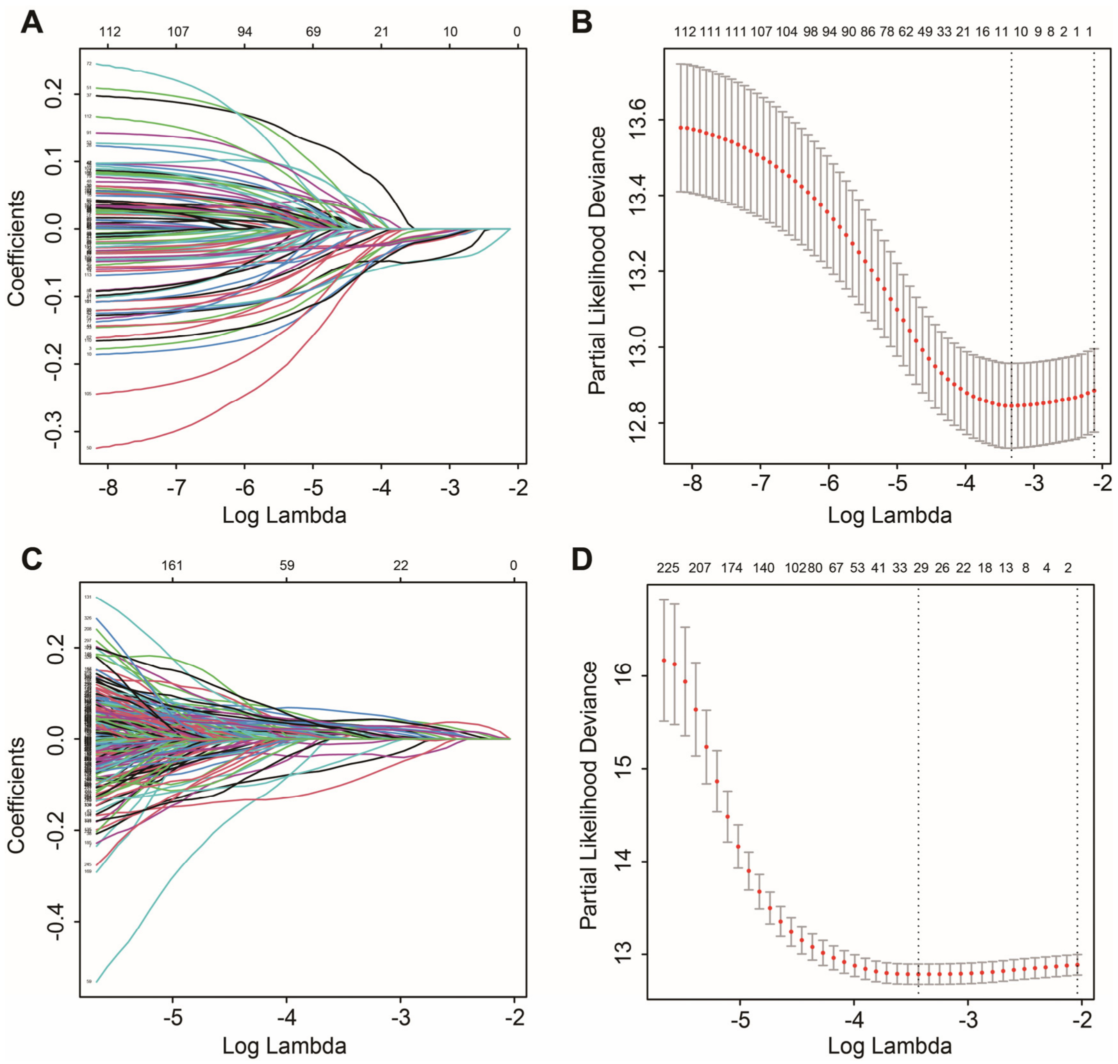

Figure S3. Selection of candidate genes via "LASSO" algorithm. **A** LASSO coefficient profiles of 114 prognostic genes in signature A. **B** Partial likelihood deviance of prognostic genes in signature A. **C** LASSO coefficient profiles of 340 prognostic genes in signature B. **D** Partial likelihood deviance of prognostic genes in signature B.
